# Nutrient trade‐offs mediated by ectomycorrhizal strategies in plants: Evidence from an *Abies* species in subalpine forests

**DOI:** 10.1002/ece3.7417

**Published:** 2021-03-16

**Authors:** Lulu Chen, Chao Jiang, Xiangping Wang, Qiuhong Feng, Xingliang Liu, Zuoxin Tang, Osbert Jianxin Sun

**Affiliations:** ^1^ School of Ecology and Nature Conservation Beijing Forestry University Beijing China; ^2^ Institute of Forestry and Climate Change Research Beijing Forestry University Beijing China; ^3^ Sichuan Wolong Forest Ecosystem Research Station Sichuan Academy of Forestry Chengdu China; ^4^ Ecological Restoration and Conservation on Forest and Wetland Key Laboratory of Sichuan Province Sichuan Academy of Forestry Chengdu China; ^5^ College of Agricultural and Life Sciences Kunming University Kunming China

**Keywords:** *Abies faxoniana*, ectomycorrhizal morphology, ectomycorrhizal strategy, plant N, plant P, plant N:P ratio, soil exploration types

## Abstract

1. Ectomycorrhizal (ECM) symbiosis is an evolutionary biological trait of higher plants for effective nutrient uptakes. However, little is known that how the formation and morphological differentiations of ECM roots mediate the nutrients of below‐ and aboveground plant tissues and the balance among nutrient elements across environmental gradients. Here, we investigated the effects of ECM foraging strategies on root and foliar N and P concentrations and N:P ratio *Abies faxoniana* under variations of climate and soil conditions.
2. The ECM symbionts preferentially mediated P uptake under both N and P limitations. The uptake efficiency of N and P was primarily associated with the ECM root traits, for example, ECM root tip density, superficial area of ECM root tips, and the ratio of living to dead root tips, and was affected by the ECM proliferations and morphological differentiations. The tissue N and P concentrations were positively associated with the abundance of the contact exploration type and negatively with that of the short‐distance exploration type.
3. Our findings indicate that the nutritional status of both below‐ and aboveground plant tissues can be strongly affected by ECM symbiosis in natural environments. Variations in the ECM strategies in response to varying environmental conditions significantly influence plant nutrient uptakes and trade‐offs.

## Graphical abstract

We studied how ECM strategies determine the partiality of N and/or P nutrition of the below‐ and aboveground in *Abies faxoniana* under both N and P deficiency and how the strategies regulated the nutrient uptakes. Contrary to findings in controlled experimental studies, here we found that the extension of ECM emanates did not always help improve the root and foliar N and P concentrations in host trees across natural environmental gradients, suggesting that the variations in the ECM strategies in *A. faxoniana* are likely driven by nutrient limitations rather than facilitating nutrient uptakes firstly in natural ecosystems.

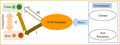

## 1 INTRODUCTION

Over 80% of tree species form ectomycorrhizal (ECM) symbionts, which are essential for maintenance of forest ecosystem health and effective soil nutrient uptakes by host trees (Barrett et al., 2011; Smith & Read, 2008). The ECM roots facilitate soil nutrient uptake through branching root tips and emanating hyphae. The ECM‐infested roots vary greatly in shape and structural configurations (Agerer, 1987–2006; Agerer, 1991) and are functionally differentiated in the capacity of soil exploration range. Agerer (2001) classified the ECM roots into five types based on the extent of soil exploration: the contact exploration (smooth mantle and only a few emanating hyphae), the short‐distance exploration (ECM root with a voluminous envelope of emanating hyphae but no rhizomorphs), the medium‐distance exploration (ECM root with rhizomorphs), the long‐distance exploration (ECM root with long rhizomorphs), and the pick‐a‐back exploration (ECM formed by members of the *Gomphidiaceae*). The five ECM exploration types differ in their capability of reaching out for soil resources at distances from the root tips through variations in the length of emanates (Pritsch & Garbaye, 2011; Tedersoo et al., 2012). The types and morphologies of ECM are found to respond to variations in soil and climatic conditions (Graefe et al., 2010; Ostonen et al., 2009; Rosinger et al., 2018; Toljander et al., 2006), and greatly affect the nutrient uptake capacity and efficiency of the host trees (Chen et al., 2016, 2018).

Both N and P are essential nutrient elements for plants and other organisms, but their limitations are common in terrestrial ecosystems. The uptake efficiency of N and P is dependent on the root systems with strategies adapting to environmental conditions (Chien et al., 2011; Hodge, 2004; Jackson & Caldwell, 1996). Of which, the ECM symbionts play an important role when trees undergo environmental stresses (Ahonen‐Jonnarth et al., 2000; Alonso et al., 2003) or nutrient deficiency (Almeida et al., 2019; Hajong et al., 2013). The symbionts help improve soil nutrient absorption by altering hyphae length, modifying the morphologies of root tips, or affecting microbial communities, when trees are under stresses (Boomsma & Vyn, 2008; Lõhmus et al., 2006; Ostonen et al., 2009). Under natural environmental conditions, uptakes of N and P by roots are enhanced by ECM foraging strategies in favor of extended exploration of soil resources (Ostonen et al.,^2007^, ^2011^). The important foraging strategies discovered so far include the secretion of enzymes decomposing N or P complex by ECM root tips, and facilitation of nutrient acquisition far from the root distal by extending hyphae or rhizomorphs (Courty et al., 2010; Nehls & Plassard, 2018; Pritsch & Garbaye, 2011).

ECM plants are characteristically of low foliar nutrients and high leaf mass per unit area, especially the tree species in Pinaceae and Fagaceae families (Cornelissen et al., 2001; Koele et al., 2012; Read, 1991). The intimate connections of foliar N nutrition and ECM symbiosis are widely reported (Hobbie et al., 2005; Hobbie & Hobbie, 2006; Koele et al., 2012). For instance, isotope tracing experiments provided direct evidence of the N transfers among plant tissues and mycorrhizal fungi (Hobbie & Högberg, 2012; Steven et al., 2004). Still, few studies have reported the associations between ECM traits and foliar nutrients. There are observations of the associations of root and leaf nutrient traits (Craine & Lee, 2003; Tjoelker et al., 2005) and reports of the positive correlations of N or P between roots and leaves (Güsewell, 2004; Liu et al., 2010). The mycorrhizal root systems are known to have the capability of assimilating N and P and then transferring them to shoots (Michelsen et al., 1996; Plassard & Dell, 2010; Smith & Read, 2008). Previous research has revealed the relationship of foliar N with mycorrhizal fungi, asserting that mycorrhizal associations influence the foliar N transfer (Craine et al., 2009; Hobbie & Hobbie, 2006). Controlled experiments demonstrated that the mycorrhizal symbionts affected the allocation of N and P nutrients among roots, stems, and leaves (Brandes et al., 1998; Chen et al., 2010; Johnson, 2010; Landis & Fraser, 2008; Wang et al., 2006). However, how ECM strategies mediate the below‐ and aboveground nutrients balance in plants in response to environmental changes yet remains unelucidated.

*Abies faxoniana* is an ancient species in the genus *Abies* that experienced the glacial and interglacial periods (Florin, 1963). It is a typical ECM tree species and naturally distributed from 2,700 to 3,900 m *asl*. in subalpine area of Sichuan Province, Southwest China. *A. faxoniana* forest is the primary vegetation type in that subalpine ecosystem. In this study, we investigated the effects of ECM strategies on the N and P nutrient uptake between below‐ and aboveground tissues in plants under different environmental gradients, that is, varying mean annual temperatures, mean annual rainfall, elevations, and soil types. The root and foliar N and P contents, ECM traits representing nutrient uptake pathway, and efficiency were measured. Our objective was to determine how the ECM strategies in *A. faxoniana* regulated the nutrient preference of N and P nutrition in below‐ and aboveground tissues. We hypothesized that (a) ECM strategies mediate the partiality of N and P nutrition in below‐ and aboveground tissues in *A. faxoniana* in response to environmental variations, and (b) ECM soil exploration types differentially regulate the nutrient uptakes in host trees.

## 2 MATERIALS AND METHODS

### 2.1 Study sites

*Abies faxoniana* is exclusively distributed in the western Sichuan Province of China, from 30°N to 35°N in latitude. Our study covered three sites along the latitudinal gradient, including the Wolong Nature Reserve (latitude 30°53′N, longitude 102°58′E), Miyaluo Nature Reserve (latitude 31°42′N, longitude 102°46′E), and Wanglang Nature Reserve (latitude 33°00′N, longitude 104°01′E).

Wolong Nature Reserve is located in the western Sichuan Plateau, with subtropical semihumid climate, and characterized by dry, cold winters and wet, cool summers (Li et al., 2020). The mean annual temperature is 4.06°C, and the mean annual precipitation is about 1,062.8 mm. The dominant woody plant species consist of *A. faxoniana*, *Picea purpurea* Mast, *Betula albosinensis* Burkill, *Betula platyphylla* Suk, *Ribes tenue* Jancz, *Sorbus koehneana* Schneid, *Rosa moyesii*, etc. The soil type is classified as dark brown soil in coniferous forest according to Chinese Soil Taxonomy (Zhang, 1983), which is developed from the weathering of slate of metamorphic rock (Taylor et al., 2006).

Miyaluo Nature Reserve is also located in the western Sichuan Plateau, with subtropical semihumid climate, and characterized by dry, cold winters and wet, cool summers (Li et al., 2013). The mean annual temperature is 1.67°C, and the mean annual precipitation is 975.2 mm. The dominant tree species consists of *A. faxoniana*, *Abies fabri* (Mast) Craib, *Picea purpurea* Mast, *Picea asperata* Mast, *Populus davidiana* Dode, *Quercus aquifolioides*, etc. The main soil type is classified as dark brown soil in coniferous forest according to Chinese Soil Taxonomy, developed from the parental materials of phyllite, slate, and schist (Keyimu et al., 2020).

Wanglang Nature Reserve is located in the Himalayas–Hengduanshan Mountains, with subtropical semihumid climate, and characterized by dry, cold winters and wet, cool summers (Zhao et al., 2012). The mean annual temperature is 4.17°C, and the mean annual precipitation is 1,021.8 mm. The dominant tree species consists of *A. faxoniana, Picea purpurea* Mast*, Sabina saltuaria, Sabina squamata, Betula albosinensis* Burkill, etc. The main soil type is classified as dark brown soil in coniferous forest according to Chinese Soil Taxonomy, with limestone as the main parental material (Taylor et al., 2006).

### 2.2 Field sampling and processing

Root and soil samples were collected during June–August 2018, at five elevations (2,850, 3,000, 3,194, 3,413, and 3,593 m *asl*.) in Wolong Nature Reserve, and two elevations in Miyaluo Nature Reserve (3,077 and 3,612 m *asl*.) and Wanglang Nature Reserve (3,070 and 3,150 m *asl*.), respectively, using point‐centered quarter sampling method (Mitchell, 2007), with randomly selected mature *A. faxoniana* trees (*n* = 8 focal trees per site) as center points. Four trees with diameter at breast height (DBH) of 35–60 cm were sampled at each center point; these trees were all within 10 m distance from the focal tree. A 10 × 10 × 10 cm soil block was collected near the lateral root at 1 m away from each target tree after clearing the surface litter. Fine roots (diameter < 2 mm) were carefully separated from the soil, and then, the soil samples from the same target tree were mixed to form a single composite sample, with a total of eight soil samples at each elevation on each site for chemical analysis. The field‐collected root and soil samples were immediately placed in zip‐lock plastic bags and stored in a cooler before being transported to laboratory for later processing. In laboratory, two random root samples for each center point were gently cleaned–washed with deionized water and stored in 5% glycerin at −20°C for ECM identification following the method of Köhle et al., (2018), and the other two samples were oven‐dried at 48°C to constant weight and used for biomass measurement and chemical analysis. Fresh soil samples were frozen‐stored at −20°C until further processing and analysis.

We collected fully developed, current‐year leaves from each target tree evenly at northern, eastern, southern, and western directions and among the four target trees at each center point. The leaf samples for each center point were mixed and placed in envelope, and then stored in a cooler before being transported to laboratory for later processing. After clean–wash with deionized water, the foliar samples were oven‐dried at 48°C until constant weight for chemical analysis.

### 2.3 ECM identification and classification

Root samples prepared in 5% glycerin were gently washed in running tap water, and soil particles adhering to root tips were removed with fine forceps under a stereoscopic microscope. When roots were covered by fungal mantles, they were classified as ectomycorrhiza. The morphology of ECM was determined under a photographic stereo microscope (Leica, M205FA, Germany), and the macroscopic and microscopic characteristics of the mycorrhizae were identified based on Agerer (1987–2006) (i.e., ECM system, color, mantle surface structure, cystidia, emanating hyphae, and rhizomorphs). The living and dead root tips were distinguished by discerning the freshness or elasticity of the root tips during the microscope observation, and the tip numbers of living and dead root tips in each soil block were counted and the ratio of living to dead root tips (Root‐tips_ratio_) was calculated. For representative ECM root samples of each morphotype in each soil block, three root tips were used for diameter (*d*, mm) and length (*l*, mm) measurements with the photographic stereo microscope. The total root tip number in each soil block was counted and identified by ECM morphotypes. The morphology diversity of ECM root tips (MDI) was measured by Simpson's index of diversity as in Lande (1996) and Matsuda and Hijii (2004); the ECM colonization ratio (C_ratio_) was measured as the percentage of the infected root tips over the total root tips. The ECM root tips per unit root biomass (ECM_tips_) were also measured. The superficial area of ECM root tips (SA) was measured for samples in each soil block, with root tips determined as a combination of cylinder and hemisphere by:

$$\text{SA}\left(m^{2}m^{‐3}\right)=\sum_{\left(i=1\right)}^{N}\left[\left(2\pi {\left(\frac{\mathrm{di}}{2}\right)}^{2}+\pi d_{i}\left(l_{i}‐d_{i}\right)\right)\times n_{i}\right]\times 10^{3}$$

where *d_i_* represents the average diameter of the ECM root tips of morphotype *i*; *l_i_* represents the average length of the ECM root tips of morphotype *i*; *n_i_* represents the number of ECM root tips of morphotype *i*; and N represents the total number of ECM morphology types.

We used the classification of Agerer (2001) and the Information System for Characterization and Determination of Ectomycorrhizae (DEEMY) database (http://www.deemy.de/) to assess the nutrient uptake strategies of ECM roots through exploration types. The ECM exploration types associated with *A. faxoniana* were categorized into contact exploration (CE), short‐distance exploration (SDE), and medium‐distance exploration (MDE) by the morphology types of ECM roots photographed with a stereo microscope. The CE type is described by the ECM roots with a smooth mantle and only a few emanating hyphae of negligible length, SDE by the ECM roots with a voluminous envelope of emanating hyphae of 0–1 mm in length but no rhizomorphs, and MDE by the ECM roots formed with rhizomorphs of 0.1–1 cm emanates. The frequency of ECM occurrence in each exploration type was calculated as the number of root tips of the specific type over the total root tip number.

### 2.4 Chemical analyses

The oven‐dried leaves and roots were analyzed for N and P concentrations on composite sampling by the central point. N concentration was measured by the elemental analyzer (vario EL III, CHNOS Elemental Analyzer; Elementar Analysensysteme GmbH, Germany). P concentration was measured by ICP (ICAP6300). The ratios of N to P in leaves and roots were calculated.

The soil samples were analyzed for pH, water content (SWC), total *N* (TN), total P (TP), available *N* (AP), ammonium *N* (${\text{NH}}_{4}^{+}$‐N), and nitrate *N* (${\text{NO}}_{3}^{‐}$‐N). Soil pH was measured with a pH meter (HI‐9125; Hanna Instruments Inc, Woonsocket, RI) by mixing the air‐dried soil sample with deionized water at a 1:2.5 ratio (w:v). SWC (%) was calculated from the mass loss after drying the fresh soil samples at 75°C to a constant weight, for at least 48 hr. TN content was analyzed using the Kjeldahl digestion procedure (Gallaher et al., 1976). TP was measured by ICP (ICAP6300). The AP, ${\text{NH}}_{4}^{+}$‐N, and ${\text{NO}}_{3}^{‐}$‐N were determined by a continuous flow analyzer (SEAL AA3, Norderstedt, Germany). Soil organic C (SOC) content was measured by a K_2_Cr_2_O_7_‐H_2_SO_4_ calefaction method (Nelson & Sommers, 1982). Soil C:N ratio (C:N_soil_) was calculated by SOC and TN. The measurements of TN, TP, and AP were all made on air‐dried soil samples, and the ${\text{NH}}_{4}^{+}$‐N and ${\text{NO}}_{3}^{‐}$‐N contents were made on fresh soil samples.

The activities of acid phosphatase (ACP) and protease (PR) were measured on the frozen‐stored soil samples. ACP was determined with *p*‐nitrophenol as a substrate (Schinner et al., 1996), with the reaction mixture of 1 g fresh soil in 1 ml 100 mM *p*‐nitrophenol. PR was determined with casein as a substrate according to Ladd and Butler (1972), with the reaction mixture of 1 g fresh soil in 5 ml casein solution (2%, w/v). The enzyme activities were expressed as µmol/g soil h^−1^.

### 2.5 Climatic data

The gridded daily climate dataset (CN05.1), with a spatial resolution of 0.25° × 0.25° constructed by the “anomaly approach” during the interpolation with more 2,400 station observations in China, was employed to obtain the meteorological data for the study sites during 1997–2016 (New et al., 2000; Xu et al., 2009). In the “anomaly approach,” we derived the final dataset by calculating a gridded climatology, then adding a gridded daily anomaly to the climatology. The air temperature for each elevation on each site was derived through topographic correction with the lapse rate of air temperature set at 0.65°C (100 m)^−1^ (Zhao et al., 2008). The same value of precipitation was assumed along the elevational gradient on each site. The temperature variables included the mean annual temperature (MAT), the mean temperature in the growing season (T_g_), the annual mean maximum air temperature (T_max_), and the annual mean maximum air temperature (T_min_); the precipitation variables included the mean annual precipitation (MAP) and the mean precipitation in the growing season (P_g_).

### 2.6 Statistical analyses

Descriptive statistical analysis of the changes in root and foliar concentrations of N and P and N:P ratios was performed by SPSS 17.0. Coefficient of variation (CV) across three study sites was calculated. Plant nutrient data were tested for normality of distribution using the Lilliefors and Shapiro–Wilk tests, and the homogeneity of variance was tested using *F* and Levene's tests. Multiple comparisons of plant nutrient variables were carried out using LSD's test for unequal sample sizes with 95% confidence intervals.

The “Varpart” function in the “Vegan” package was used to partition the variation of root and foliar nutrition traits (N and P contents, and N:P ratio) into components under three categories of predictors (i.e., soil chemistry factors, climatic factors, and ECM trait factors) in RStudio.

By loading the “lavaan()” function in RStudio, we performed a structural equation models (SEMs) among soil variables, ECM traits and plant nutrients (N and P contents, and N:P ratio). Firstly, we identified key soil variables and ECM traits by principal component analysis (PCA), and the first and second principal components of soil variables (named Soil‐PC1 and Soil‐PC2, respectively) and ECM traits (named ECM‐PC1 and ECM‐PC2, respectively) were selected for SEMs analyses. Then, we constructed SEMs for the effects of soil and ECM PC axes and climate factors (MAT, MAP) on root and foliar nutrients (i.e., concentrations of N and P and N:P ratio). Redundancy analysis (RDA) was used to determine the relationships of plant nutrient traits and ECM root traits across all sites and elevations. Hellinger or standardized transformation was used to transform the data of plant nutrient traits and ECM root traits for RDA.

Curve estimation models were used to estimate the relationships of root and foliar nutrient variables with the colonization ratio of the soil exploration types (*n* = 9). Of which, data of the colonization ratio of MDE and root P were transformed by SQRT for the regression analysis.

## 3 RESULTS

### 3.1 Variations in root and foliar N and P

The foliar N and P concentrations were most variable, and root N and P concentrations were least variable, across the three study sites and along the elevational gradient, as inferred by the CV values (Table 1). The mean root N concentration was significantly lower (*p* < .05) than the mean foliar N concentration, while the root N:P ratio was significantly higher (*p* < .05) than the foliar N:P ratio, across sites and elevations.

**TABLE 1:** Descriptive statistics of root and foliar N and P nutrients in *Abies faxoniana* across the three study sites

Statistical parameter	Foliar N:P	Root N:P	Foliar N (mg/g)	Foliar P (mg/g)	Root N (mg/g)	Root P (mg/g)
*N*	72	72	72	72	72	72
Mean	8.47b	15.40a	17.44a	2.24c	10.81b	0.72c
*SD*	1.86	3.85	6.98	1.22	2.06	0.13
CV	21.97%	25.01%	40.02%	54.76%	19.05%	17.43%

Note

Different letters designate significant difference among nutrient variables at 0.05 level.

**FIGURE 1:**
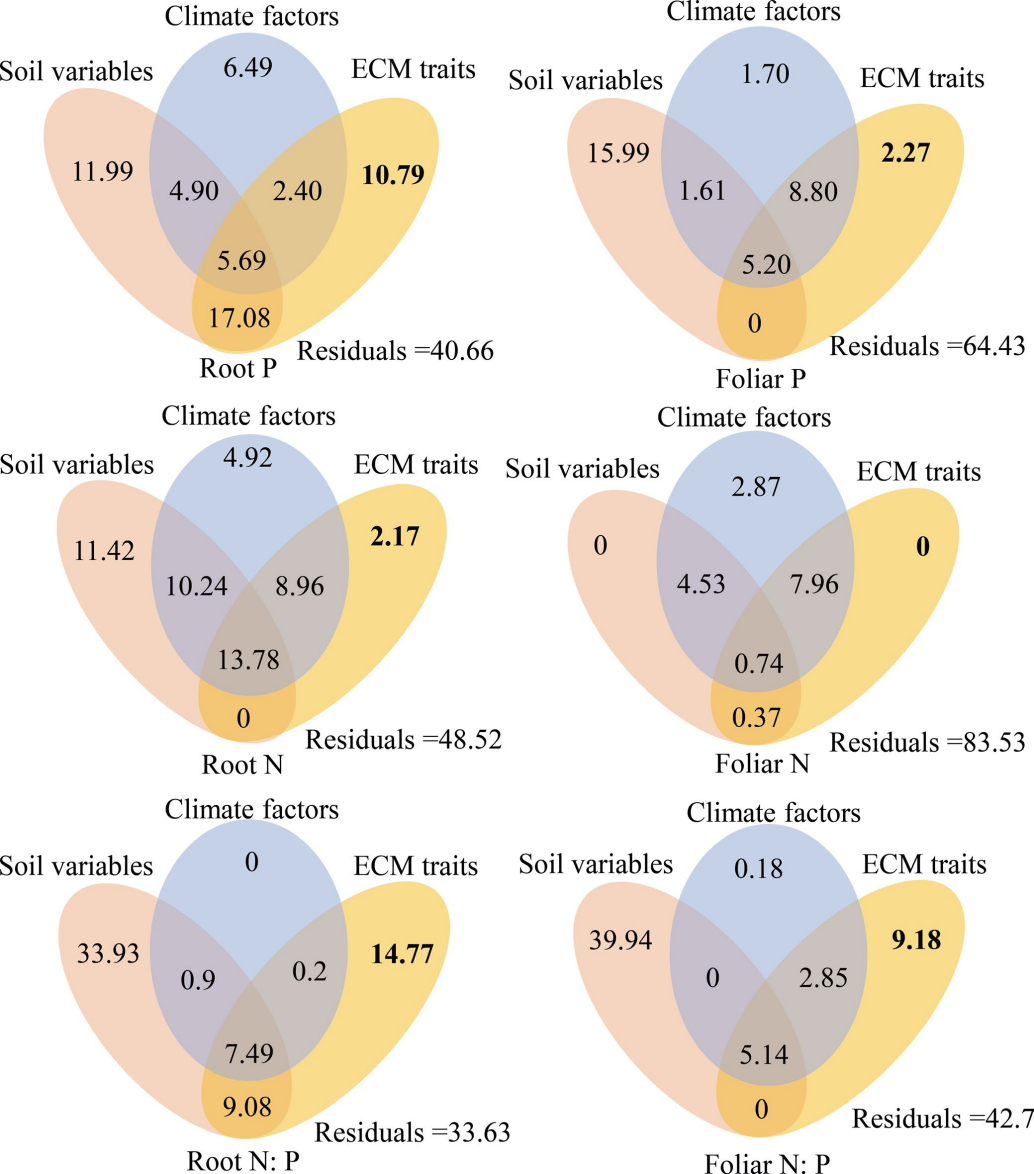
Schematic diagram of variation partitioning in determining the effects of soil variables, climate factors, and ECM traits on root and foliar nutrient variables. Values in diagram represent the explained variations in each category of factors and various interactions

Variance partitioning shows that the root N and P concentrations were predominantly influenced by soil factors (Figure 1), with the root P concentration further affected by ECM traits with an explained variance of 10.8%. The variance in foliar N was mostly explained by the joint effect of the climate factors and the ECM traits. Among the different categories of factors, soil environmental conditions were most influential on root and foliar N:P ratios, explaining 33.9% and 39.9% of the variations, respectively, while the ECM traits were secondary in affecting the root and foliar N:P ratios, explaining 14.8% and 9.18% of the variations, respectively.

### 3.2 Influences of ECM traits on root and foliar N and P

The results of PCA on soil variables show that PC1 and PC2, respectively, explained 39.8% and 14.9% of the variations; the variables with high scores include ACP, pH, TN, ${\text{NO}}_{3}^{‐}$‐N, TP, and SWC on PC1, and PR, C:N_soil_, ${\text{NH}}_{4}^{+}$‐N, and AP on PC2 (Table 2). For the ECM traits, PC1 and PC2, respectively, explained 40.4% and 17.8% of the variations; variables with high scores include C_ratio_, MDI, FRB, CE, SDE, SA, Root‐tips_ratio_, and ECM_tips_ on PC1, and CE, MDE, and Root‐tips_ratio_ on PC2 (Table 2).

**TABLE 2:** Scores of factors on PC1 and PC2 in the soil variables and ECM traits based on principal component analysis (PCA)

	Factors	PC1	PC2		Traits	PC1	PC2
Soil variables	SWC	1.52	−0.19	ECM traits	FRB	−0.78	−0.09
	pH	0.89	0.43		Root‐tips_ratio_	1.31	−0.73
	TN	1.49	−0.15		C_ratio_	1.4	−0.55
	C:N_soil_	0.13	−0.76		ECM_tips_	0.88	0.43
	NH_4_^+^‐*N*	0.62	−1.09		CE	1.24	0.89
	${\text{NO}}_{3}^{‐}$‐*N*	1.22	0.4		SDE	−0.8	−0.43
	PR	0.56	0.76		MDE	0.22	−1.49
	TP	0.88	0.06		MDI	−1.4	−0.37
	AP	−0.12	−1.09		SA	0.97	−0.37
	ACP	1.51	−0.14				

Abbreviations: ${\text{NH}}_{4}^{+}$‐N, ammonium nitrogen; ${\text{NO}}_{3}^{‐}$‐N, nitrate nitrogen; ACP, soil acid phosphatase; AP, available phosphorus; C:N_soil_, Soil C:N ratio; CE, ECM roots of contact exploration type; C_ratio_, Colonization ratio of ECM fungi; ECM_tips_, ECM root tips per unit root biomass; FRB, fine root biomass; MDE, ECM roots of medium‐distance exploration type; MDI, morphology diversity index; PR, soil protease; Root‐tips_ratio_, the ratio of the living to dead root tips; SA, superficial area of ECM; SDE, ECM roots of short‐distance exploration type; SWC, soil water content; TN, total nitrogen; TP, total phosphorus.

**FIGURE 2:**
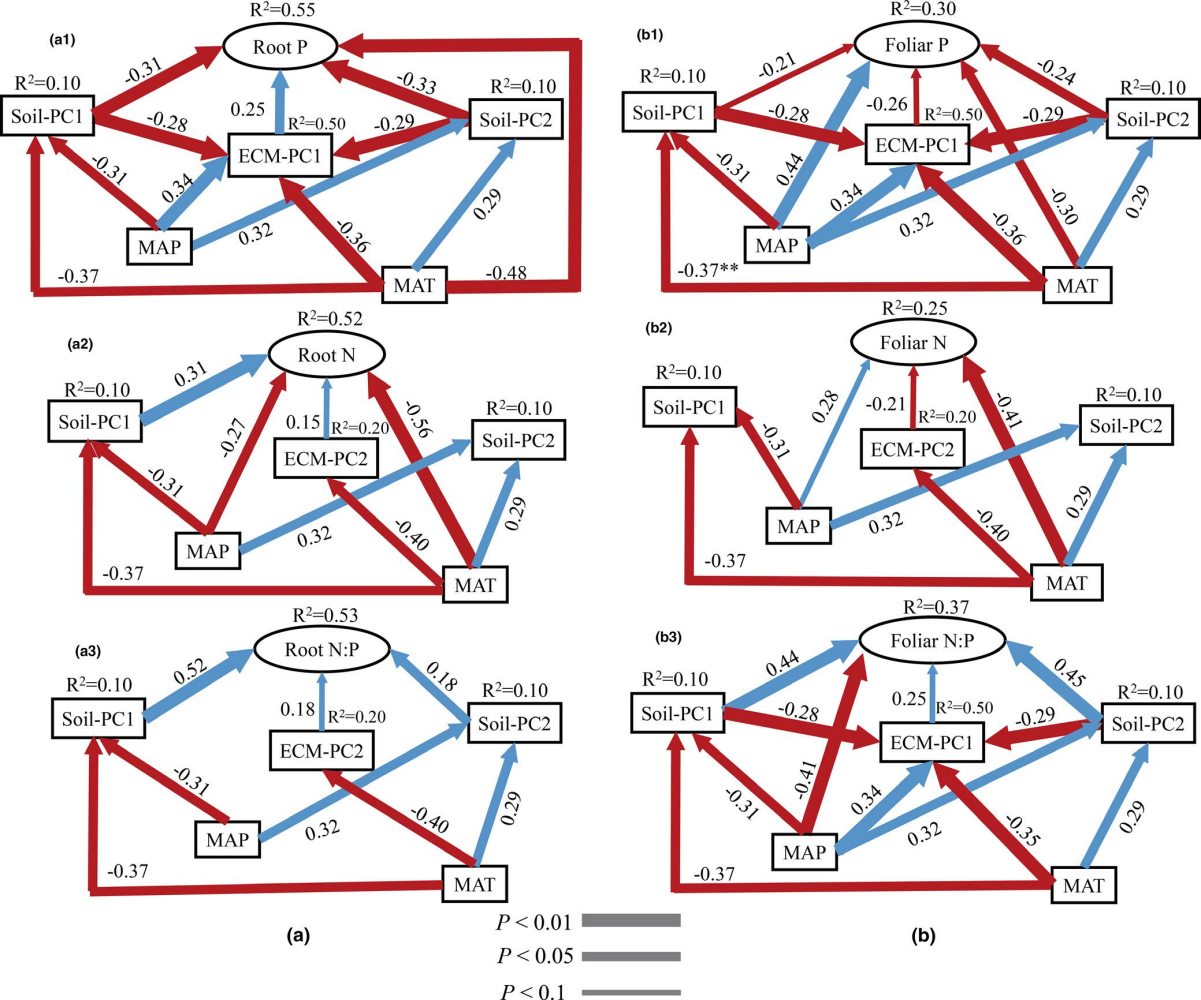
Analysis of structural equation models (SEMs) on root and foliar nutrient variables. Standardized path coefficients (δ) are displayed on line arrows; significant paths are color‐coded in blue if positive, and in red, if negative. *R* ^2^ value represents the proportion of total variance explained for the specific dependent variable. (a1): SEMs depicting the regulatory pathway of the controls on root P; (a2): SEMs depicting the regulatory pathway of the controls on root N; (a3): SEMs depicting the regulatory pathway of the controls on root N:P; (b1): SEMs depicting the regulatory pathway of the controls on foliar P; (b2): SEMs depicting the regulatory pathway of the controls on foliar N; (b3)**:** SEMs depicting the regulatory pathway of the controls on foliar N:P. ECM‐PC1: the first principal components of ECM traits by PCA; ECM‐PC2: the second principal components of ECM traits by PCA. MAT: the mean annual temperature; MAP: the mean annual precipitation. Soi‐PC1: the first principal components of soil factors principal component analysis (PCA); Soi‐PC2: the second principal components of soil factors by PCA

The SEMs illustrate that the ECM traits affected root and foliar nutrient variables, in addition to the direct and significant effects of soil variables and climate factors (Figure 2). Root nutrients were positively affected the ECM traits, while the foliar nutrients, with exception of foliar N:P, were negatively affected. The SEMs explained 55% of the variance in root N concentration, 52% in root P concentration, and 53% in root N:P ratio, respectively. ECM‐PC1 had a significant and positive correlation (*p* < .05) with root P concentration, and a marginal correlation (*p* < .1) with root N concentration and N:P ratio (Figure 2a1–3). The variances explainable by SEMs in foliar N concentration, P concentration, and N:P ratio were at 30, 25, and 37%, respectively. Both foliar P concentration and the N:P ratio were marginally correlated (*p* < .1) with ECM‐PC1, while foliar N concentration was marginally correlated (*p* < .1) with ECM‐PC2 (Figure 2b1–3).

The soil variables and climate factors also imposed indirect effects on root and foliar nutrients by influencing ECM traits. While root and foliar P concentrations and foliar N:P ratio were significantly affected by ECM‐PC1, this component was significantly affected by soil‐PC1, soil‐PC2, MAT, and MAP (Figure 2). MAT had a significant effect on ECM‐PC2, which in turn significantly affected root and foliar N concentrations and root N:P ratio.

**FIGURE 3:**
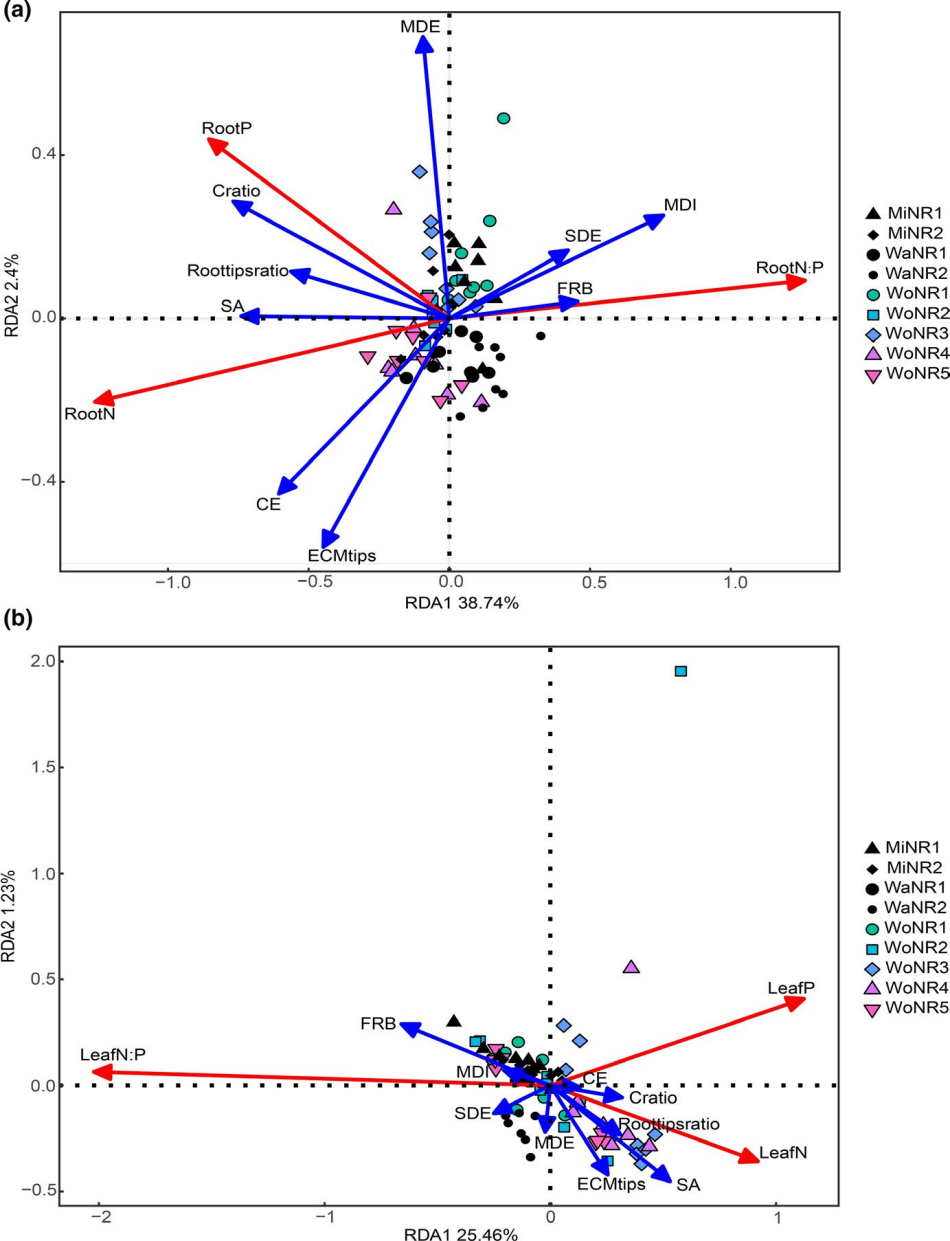
Redundancy analysis (RDA) ordination biplot of ECM traits and root nutrients (a) and foliar nutrients (b). C_ratio_: Colonization ratio of ECM fungi; CE: ECM roots of contact exploration type; ECM_tips_: ECM root tips per unit root biomass; FRB: fine root biomass; MDE: ECM roots of medium‐distance exploration type; MDI: morphology diversity index; Root‐tips_ratio_: the ratio of the living to dead root tips; SA: superficial area of ECM; SDE: ECM roots of short‐distance exploration type; MiNR1: sampling at 3,077 m *asl* in Miyaluo Nature Reserve; MiNR2: sampling at 3,612 m *asl* in Miyaluo Nature Reserve; WaNR1: sampling at 3,070 m *asl*. in Wanglang Nature Reserve; WaNR2: sampling at 3,150 m *asl* in Wanglang Nature Reserve; WoNR1: sampling at 2,850 m *asl* in Wolong Nature Reserve; WoNR2: sampling at 3,000 m *asl* in Wolong Nature Reserve; WoNR3: sampling at 3,194 m *asl* in Wolong Nature Reserve; WoNR4: sampling at 3,413 m *asl* in Wolong Nature Reserve; WoNR5: sampling at 3,593 m *asl* in Wolong Nature Reserve

The RDA axis 1 (RDA1) and axis 2 (RDA2), respectively, explained 38.7% and 2.4% of the variations in root nutrients, and 25.5% and 1.23% of the variations in foliar nutrients (Figure 3). There were significant positive relationships of root P concentration with C_ratio_ and Root‐tips_ratio_, between root N concentration and SA, and of the root N:P ratio with FRB and MDI; both root N and P concentrations were negatively correlated to the SDE, MDI, and FRB (Figure 3a), whereas in leaves, there were significant positive relationships between P concentration and CE, of N concentration with C_ratio_, Root‐tips_ratio_, SA, and CE, and of the N:P ratio with FRB and MDI, respectively. The foliar N concentration was negatively correlated to SDE, MDI, and FRB, and the foliar P was negatively correlated to SDE, MDE MDI, and FRB (Figure 3b).

### 3.3 Relationships of root and foliar nutrients with soil exploration types

**FIGURE 4:**
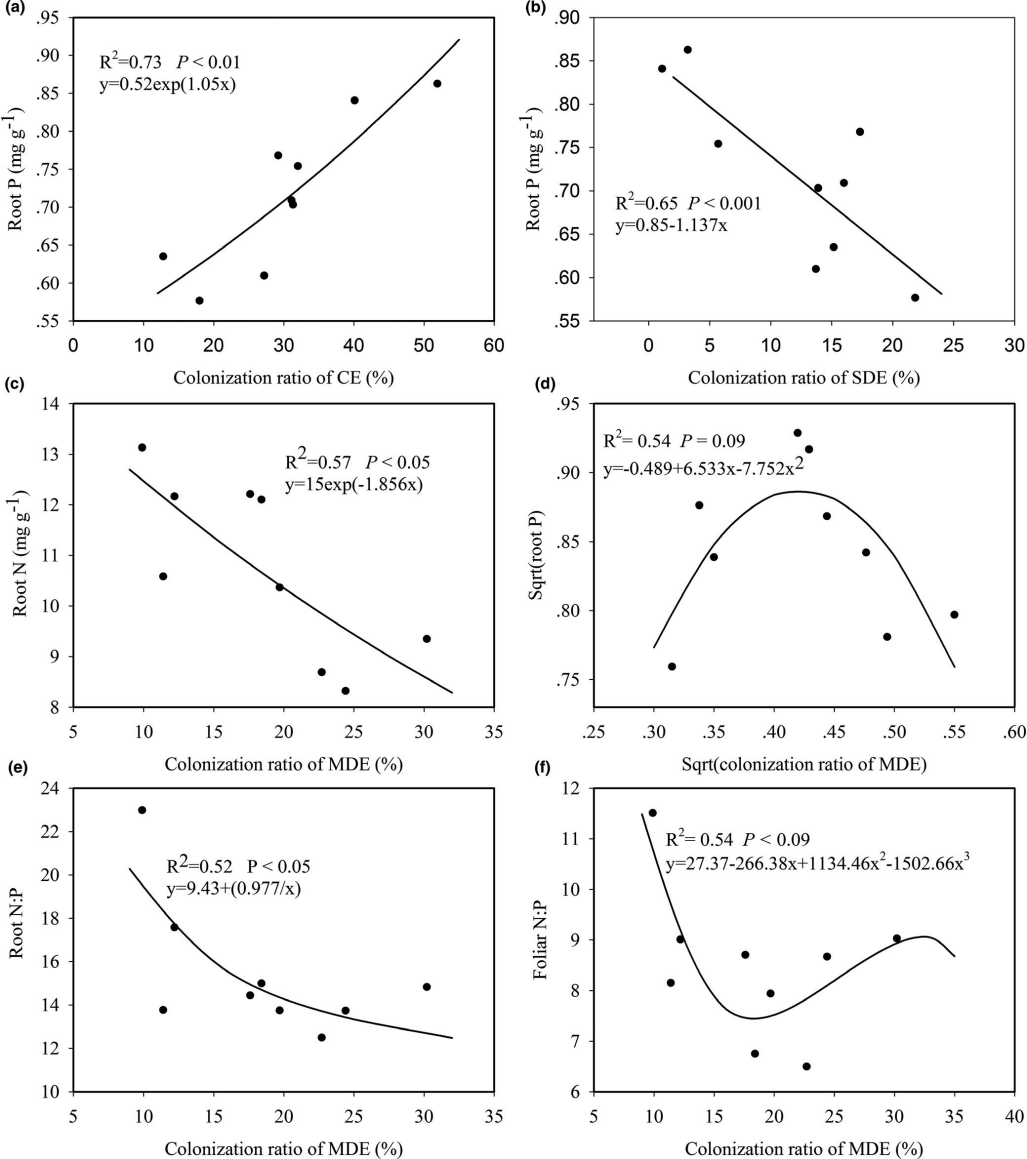
The significant relationships of root and foliar nutrients with soil exploration types. (a): the regressions of root P and the colonization ratio of CE; (b): the regressions of root P and the colonization ratio of SDE; (c): the regressions of root N and the colonization ratio of SDE; (d): the regressions of sqrt(root P) and sqrt(the colonization ratio of MDE); (e): the regressions of root N:P and the colonization ratio of MDE; (f): the regressions of foliar N:P and the colonization ratio of MDE. CE: ECM roots of contact exploration type; SDE: ECM roots of short‐distance exploration type; MDE: ECM roots of medium‐distance exploration type

Root P concentration was positively related to the colonization ratio of CE with an exponential relationship (*R* ^2^ = 0.73, *p* < .01; Figure 4a). Root P and N concentrations were negatively related to the colonization ratio of SDE with a linear (*R* ^2^ = 0.57, *p* < .05; Figure 4b) and exponential relationships (*R* ^2^ = 0.65 *p* < .001; Figure 4c), respectively. As illustrated in Figure 4d, root P concentration varied with the colonization ratio of MDE in a curvilinear relationship, and increased firstly with the increase in the colonization ratio of MDE, then decreased with the increase in MDE. Root N:P ratio decreased with the increase in the colonization ratio of MDE (*R* ^2^ = 0.52, *p* < .05; Figure 4e). The foliar N:P ratio decreased with the colonization ratio of MDE at values < 20%, increased at values > 20% (*R* ^2^ = 0.56, *p* < .09; Figure 4f).

## 4 DISCUSSION

**FIGURE 5:**
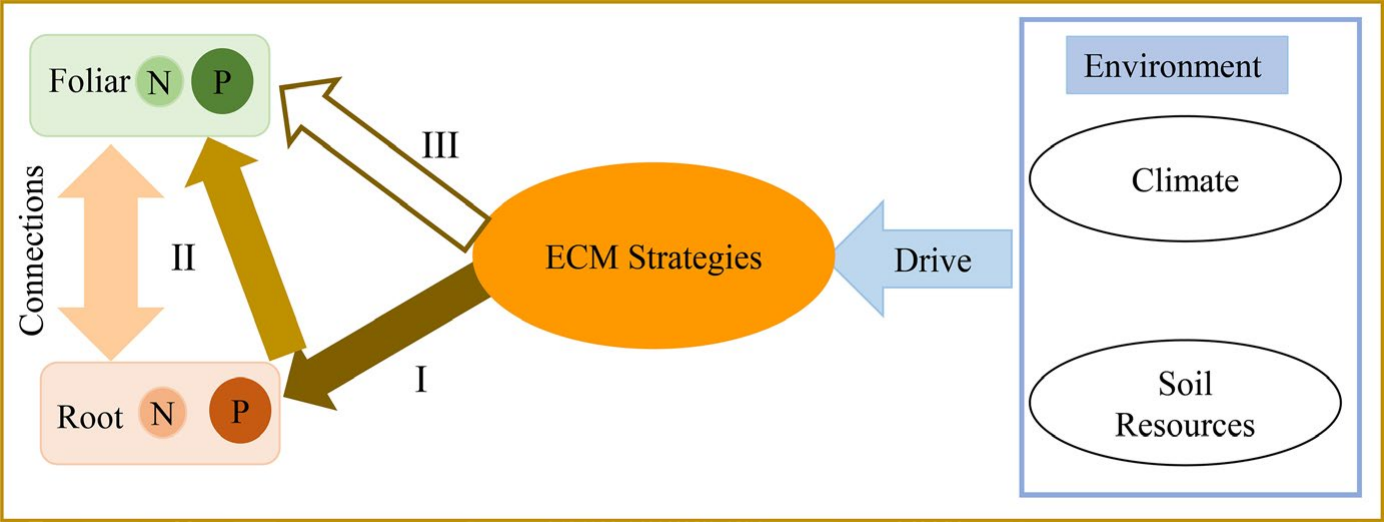
A conceptual model of the intervention of ECM symbiosis on root and foliar N and P in *Abies faxoniana*. I: The primary effects of ECM symbiosis on root nutrients. Root N and P nutrients are both strongly affected by ECM symbiosis, but the effects are stronger on root P than root N; II: indirect mediation of ECM symbiosis on foliar N and P driven by the nutrient limitation signals from leaves to roots; III: changes in foliar N and P caused by variations in ECM strategies. Changes in ECM foraging strategies impose greater influences on foliar P than on foliar N

It is widely recognized that ECM fungi promote the uptake of N and P in plants. While the root N and P nutrition of ECM plants has been widely studied (Almeida et al., 2019; Franklin et al., 2014; Zhang et al., 2019), relatively few studies have attempted to determine the attributions of ECM symbionts to aboveground nutrition in tree species (Koele et al., 2012; Michelsen et al., 1996). In this study, we examined the effects of the variations in ECM symbiosis on root and foliar N and P nutrition in *A. faxoniana* under varying soil types and climate factors. Generally, the ECM in *A. faxoniana* appeared to be more important in P uptake than N uptake under both N and P limitations (Figures 1, 2, and 5). The ECM traits in *A. faxoniana* were better correlated with root N and P concentrations than with the foliar N and P concentrations (Figure 2). The ECM soil exploration types exerted differential impacts on root and foliar N concentrations and N:P ratio (Figure 4).

### 4.1 Differential effects of ectomycorrhizal strategies on the below‐ and aboveground plant N and P nutrients

Concerning our first hypothesis that ECM strategies mediate the partiality of N and P nutrition in below‐ and aboveground tissues in *A. faxoniana* in response to environmental variations, we found distinct effects of ECM strategies on plant N and P elemental stoichiometry in roots and leaves under varying soil and climate conditions. In this study, the mature *A. faxoniana* trees were deficient in root P (Table 1, P concentration: 0.72 ± 0.13 mg/g) as well as in root and foliar *N* (values of N concentration < 10 mg/g and N:P ratio < 14). It is suggested that the values of N:P ratio < 14 or >16, respectively, indicate N limitation or P limitation in plants and that tissue P concentration < 1 mg/g and tissue N concentration < 10 mg/g are considered as deficient of the nutrient (Güsewell, 2004; Güsewell & Koerselman, 2002; Tessier & Raynal, 2003). P is generally more limiting than N in terrestrial ecosystems as it is derived primarily from rock weathering and uniquely depended on root systems (Vitousek et al., 2010; Walker & Syers, 1976). According to the results in this study, the variations of ECM traits in *A. faxoniana* affected more on root and foliar P concentrations than on N concentrations (Figures 1 and 2), suggesting that ECM strategies are more functional on P uptakes than on N uptakes under both N and P limitations. Basically, the resource allocation in belowground by the mycorrhizal symbiosis is expected to abide by the nutrient requirements of plants (Merrild et al., 2013). However, the priority in nutrient acquisition is frequently determined by the strategic choices of plant species under multiple element limitations. It has been demonstrated that the ECM symbiosis give priority to the uptake of P but not N when in deficient supplies under different experimental conditions (Almeida et al., 2019; Smith et al., 2011; Zavišić et al., 2016). Moreover, it has been reported that the ECM symbioses sometimes do not largely alleviate N limitation (Franklin et al., 2014; Näsholm et al., 2013) and that plants could obtain N by the root pathway rather than the mycorrhizal symbioses which would require extra C investment under N shortage (Jiang et al., 2017; Zhang et al., 2019).

The differential mechanisms of nutrient acquisition might change the nutrition preferences in plant species (Houlton et al., 2007; Zhang et al., 2018). Apart from soil resources and climate factors, our study shows that the varied ECM traits greatly influenced N and P nutrients in *A. faxoniana* (Figures 1 and 2). Overall, the ECM traits associated with the uptake efficiency, such as the colonization ratio of ECM root tips, the ratio of the living to dead root tips, the colonization ratio of the contact exploration type, and the superficial area of ECM root tips, were all positively correlated to the below‐ and aboveground N and P concentrations in *A. faxoniana* (Figure 3). However, the fine root biomass and morphological diversity of ECM roots impacted negatively on the tissue N and P concentrations but positively on the N:P ratio, suggesting the trade‐offs between the C investment for ECM root proliferation and morphology differentiation and the nutrients uptake. Accordingly, we draw the conclusion that both N and P nutrients of roots and leaves in *A. faxoniana* are primarily mediated by the nutrient uptake efficiency of ECM roots, while the N and P stoichiometry is strongly related to the alteration of uptake or transportation pathway of ECM roots. Research shows that the nutrient uptake efficiency of the symbiotic fungi in plants might mediate the concentration of the nutrients in roots and leaves, for example, the ECM colonization ratio, ECM absorption root vigor (Beltrano et al., 2013; Li et al., 2015; Vandenkoornhuyse et al., 2003), ECM root tip density, and absorptive capacity of ECM emanates (Ostonen et al., 2011), while the N and P stoichiometry in plant species could be affected by the function of mycorrhizal symbionts, for example, hyphae exploration ability and/or extracellular enzyme secretion (Chen et al., 2010). Plant nutrient uptake and balance exceedingly depend on the alternative foraging strategies of the ECM root systems (e.g., foraging precision of hyphae, morphology plasticity, and foraging range) under different environmental conditions (Chen et al., 2018; Einsmann et al., 1999; Köhle et al., 2018; Wang et al., 2006). While controlled experiments and isotope tracing studies have demonstrated that ECM symbionts contribute to the improvements of plant biomass, foliar N and P acquisition (Brandes et al., 1998; Craine et al., 2009; Hobbie & Hobbie, 2006), such functional roles are not readily observable in natural ecosystems due to the confounding effects of biotic and biotic environments. In this study, under the varying soil and climate conditions, we were able to reveal the differential roles of ECM strategies in N and P uptake in *A. faxoniana*.

### 4.2 Trade‐offs of nutrient uptake and soil exploration types in *Abies faxoniana*

In confirmation to our second hypothesis that ECM soil exploration types differentially regulate the nutrient uptakes in host trees, we found that the concentrations of root and foliar N and P in *A. faxoniana* were positively associated with the frequency of contact exploration type (Figures 2, 3a,b, and 4a) and negatively with that of the short‐distance and the medium‐distance exploration types (Figure 3a,b). Specifically, both root N and P concentrations were negatively associated with the frequency of short‐distance exploration type, while a quadratic relationship was found between the root P concentration and the frequency of the medium‐distance exploration type in a value range of 9%–30% (Figure 4b–d). Clearly, variations in the length of emanates in *A. faxoniana* in response to varying environmental conditions was not explainable by adjustment in nutrient uptake capacity. This contradicts the findings of positive relationships between the nutrient status of host plants and the length of emanates of ECM roots in literature (Agerer, 2001; Brandes et al., 1998; Hobbie & Agerer, 2010; Lilleskov et al., 2011). A probable explanation for this contradiction is that, in natural ecosystems, while ECM symbionts respond to soil resource deficiency in the way of root proliferation and production of emanating hyphae, the consequence of improved nutrition in host plants could be offset by increased energy cost for the ECM root systems under multiple resource limitation. In this study, the soil N and P were mostly deficient across the study sites (alkaline N: 40.78 ± 18.83 mg/g, total P: 0.87 ± 0.26 mg/g). It is likely that the contact exploration type and the short‐distance exploration type mediated the uptake of alkaline N and available P, whereas the medium‐distance exploration type helped forage the organic N and P far from the root distal (Agerer, 2001; Hobbie & Agerer, 2010). Ostonen et al., (2007), Ostonen et al., (2011) noted that host trees can rely on the high efficiency of resource capture of the root–mycorrhiza continuum while investing little C to ECM root systems. Besides, plants would cut down the investment when C allocation outweigh the benefit obtained from ECM fungi (Johnson et al., 2003; Treseder, 2004), or ECM fungi sometimes hold the nutrients for themselves in priority while the host tree remains nutrient deficient under extreme nutrient limitations (Treseder & Allen, 2002). The improved root and foliar N and P by the occurrence of the contact exploration type and the negative relationships with the frequency of the short‐distance and the medium‐distance exploration types may partially attribute to trade‐offs between the C allocation to ECM emanates and nutrient uptake in host plants (Johnson et al., 2013; Magyar et al., 2007).

Our findings allow us to develop a conceptual model on the intervention of ECM symbiosis on root and foliar N and P nutrition using *A. faxoniana* as a case study (Figure 5). The model illustrates that the ECM strategies strongly affect the root nutrients, and then through the interconnections between roots and aboveground tissues in nutrient transportation and re‐allocations, eventually influence the foliar nutrients, with preferential effects on P under both N and P limitations.

## CONFLICT OF INTEREST

None declared.

## AUTHOR CONTRIBUTIONS

**Lulu Chen:** Conceptualization (equal); data curation (equal); formal analysis (equal); investigation (equal); writing–original draft (equal). **Chao Jiang:** Formal analysis (equal); methodology (equal); resources (equal). **Xiangping Wang:** Resources (equal); supervision (equal); writing–review and editing (equal). **Qiuhong Feng:** Data curation (equal); resources (equal); writing–review and editing (equal). **Xingliang Liu:** Data curation (equal); resources (equal); writing–review and editing (equal). **Zuoxin Tang:** Formal analysis (equal); methodology (equal); writing–review and editing (equal). **Osbert Jianxin Sun:** Conceptualization (lead); funding acquisition (lead); methodology (equal); project administration (lead); resources (lead); supervision (lead); writing–review and editing (lead).

## ETHICAL APPROVAL

Performance of this study did not involve any critical damage to plants and ecosystems at the sites. Permissions for access to study sites and sampling were granted by the local administrations and management offices.

## Data Availability

Data supporting this research are available from the Dryad Digital Repository at: https://doi.org/10.5061/dryad.pvmcvdnkb.

## References

[ece37417-bib-0001] Agerer, R. (1987–2006). Colour Atlas of Ectomycorrhizae. : Einhorn‐verlag.

[ece37417-bib-0002] Agerer, R. (1991). Characterization of ectomycorrhiza. Methods in Microbiology, 23, 26–65. 10.1016/S0580-9517(08)70172-7

[ece37417-bib-0003] Agerer, R. (2001). Exploration types of ectomycorrhizae: A proposal to classify ectomycorrhizal mycelial system according to their patterns of differentiation and putative ecological importance. Mycorrhiza, 11, 107–114.

[ece37417-bib-0004] Ahonen‐Jonnarth, U. , van Hees, P. A. W. , Lundstrm, U. S. , & Finlay, R. D. (2000). Organic acids produced by mycorrhizal *Pinus sylvestris* exposed to elevated aluminum and heavy metal concentrations. New Phytologist, 146, 557–567. 10.1046/j.1469-8137.2000.00653.x

[ece37417-bib-0005] Almeida, J. P. , Rosenstock, N. P. , Forsmark, B. , Bergh, J. , & Wallander, H. (2019). Ectomycorrhizal community composition and function in a spruce forest transitioning between nitrogen and phosphorus limitation. Fungal Ecology, 40, 20–31. 10.1016/j.funeco.2018.05.008

[ece37417-bib-0006] Alonso, J. , García, M. A. , Pérez‐López, M. , & Melgar, M. J. (2003). The concentrations and bioconcentration factors of copper and zinc in edible mushrooms. Archives of Environmental Contamination and Toxicology, 44, 180–188. 10.1007/s00244-002-2051-0 12520390

[ece37417-bib-0007] Barrett, G. , Campbell, C. D. , Fitter, A. H. , & Hodge, A. (2011). The arbuscular mycorrhizal fungus *Glomus hoi* can capture and transfer nitrogen from organic patches to its associated host plant at low temperature. Applied Soil Ecology, 48(1), 102–105. 10.1016/j.apsoil.2011.02.002

[ece37417-bib-0008] Beltrano, J. , Ruscitti, M. , Arango, M. C. , & Ronco, M. (2013). Effects of arbuscular mycorrhiza inoculation on plant growth, biological and physiological parameters and mineral nutrition in pepper grown under different salinity and P levels. Journal of Soil Science and Plant Nutrition, 13, 123–141. 10.4067/S0718-95162013005000012

[ece37417-bib-0009] Boomsma, C. R. , & Vyn, T. J. (2008). Maize drought tolerance: Potential improvements through arbuscular mycorrhizal symbiosis? Field Crops Research, 108, 14–31. 10.1016/j.fcr.2008.03.002

[ece37417-bib-0010] Brandes, B. , Godbold, D. L. , Kuhn, A. J. , & Jentschke, G. (1998). Nitrogen and phosphorus acquisition by the mycelium of the ectomycorrhizal fungus *Paxillus involutus* and its effect on host nutrition. New Phytologist, 140, 735–743. 10.1046/j.1469-8137.1998.00313.x 33862956

[ece37417-bib-0011] Chen, M. M. , Yin, H. B. , O’Connor, P. , Wang, Y. S. , & Zhu, Y. G. (2010). C:N: P stoichiometry and specific growth rate of clover colonized by arbuscular mycorrhizal fungi. Plant and Soil, 326, 21–29. 10.1007/s11104-009-9982-4

[ece37417-bib-0012] Chen, W. L. , Koide, R. T. , Adams, T. S. , DeForest, J. L. , & Cheng, L. (2016). Root morphology and mycorrhizal symbioses together shape nutrient foraging strategies of temperate trees. Proceedings of the National Academy of Sciences of the United States of America, 113, 8741–8746. 10.1104/pp.108.117820 27432986 PMC4978252

[ece37417-bib-0013] Chen, W. L. , Koide, R. T. , & Eissenstat, D. M. (2018). Nutrient foraging by mycorrhizas: From species functional traits to ecosystem processes. Functional Ecology, 32, 858–869. 10.1111/1365-2435.13041

[ece37417-bib-0014] Chien, S. H. , Gearhart, M. M. , & Villagarcia, S. (2011). Comparison of ammonium sulfate with other nitrogen and sulfur fertilizers in increasing crop production and minimizing environmental impact: A review. Soil Science, 176, 327–335. 10.1097/SS.0b013e31821f0816

[ece37417-bib-0015] Cornelissen, J. H. C. , Aerts, R. , Cerabolini, B. , Werger, M. J. A. , & van der Heijden, M. G. A. (2001). Carbon cycling traits of plant species are linked with mycorrhizal strategy. Oecologia, 129, 611–619. 10.1007/s004420100752 24577702

[ece37417-bib-0016] Courty, P. E. , Buee, M. , Diedhiou, A. G. , Fre‐Klett, P. , Le Tacon, F. , Rineau, F. , Turpault, M. P. , Uroz, S. , & Garbaye, J. (2010). The role of ectomycorrhizal communities in forest ecosystem processes: New perspectives and emerging concepts. Soil Biology and Biochemistry, 42, 679–698. 10.1016/j.soilbio.2009.12.006

[ece37417-bib-0017] Craine, J. M. , Elmore, A. J. , Aidar, M. P. M. , Bustamante, M. , Dawson, T. E. , Hobbie, E. A. , Kahmen, A. , Mack, M. C. , McLauchlan, K. K. , Michelsen, A. , Nardoto, G. B. , Pardo, L. H. , Peñuelas, J. , Reich, P. B. , Schuur, E. A. G. , Stock, W. D. , Templer, P. H. , Virginia, R. A. , Welker, J. M. , & Wright, I. J. (2009). Global patterns of foliar nitrogen isotopes and their relationships with climate, mycorrhizal fungi, foliar nutrient concentrations, and nitrogen availability. New Phytologist, 183, 980–992. 10.1111/j.1469-8137.2009.02917.x 19563444

[ece37417-bib-0018] Craine, J. M. , & Lee, W. G. (2003). Covariation in leaf and root traits for native and non‐native grasses along an altitudinal gradient in New Zealand. Oecologia, 134, 471–478. 10.1007/s00442-002-1155-6 12647118

[ece37417-bib-0019] Einsmann, J. C. , Jones, B. , Pu, M. , & Mitchell, R. J. (1999). Nutrient foraging traits in 10 co‐occurring plant species of contrasting life forms. Journal of Ecology, 87, 609–619. 10.1046/j.1365-2745.1999.00376.x

[ece37417-bib-0020] Florin, R. (1963). The distribution of conifer and taxad genera in time and space. Acta Horti Bergiani, 20, 121–312.

[ece37417-bib-0021] Franklin, O. , Nasholm, T. , Hogberg, P. , & Hogberg, M. N. (2014). Forests trapped in nitrogen limitation – An ecological market perspective on ectomycorrhizal symbiosis. New Phytologist, 203, 657–666. 10.1111/nph.12840 PMC4199275 24824576

[ece37417-bib-0022] Gallaher, R. N. , Weldon, C. O. , & Boswell, F. C. (1976). A semi‐automated procedure for total nitrogen in plant and soil samples. Soil Science Society of America Journal, 40, 887–889. 10.2136/sssaj1976.03615995004000060026x

[ece37417-bib-0023] Graefe, S. , Hertel, D. , & Leuschner, C. H. (2010). N, P and K limitation of fine root growth along an elevation transect in tropical mountain forests. Acta Oecologica, 36, 537–542. 10.1016/j.actao.2010.07.007

[ece37417-bib-0024] Güsewell, S. (2004). N: P ratios in terrestrial plants: Variation and functional significance. New Phytologist, 164, 243–266. 10.1111/j.1469-8137.2004.01192.x 33873556

[ece37417-bib-0025] Güsewell, S. , & Koerselman, W. (2002). Variation in nitrogen and phosphorus concentrations of wetland plants. Perspectives in Ecology, Evolution and Systematics, 5, 37–61. 10.1078/1433-8319-0000022

[ece37417-bib-0026] Hajong, S. , Kumaria, S. , & Tandon, P. (2013). Comparative study of key phosphorus and nitrogen metabolizing enzymes in mycorrhizal and non‐mycorrhizal plants of *Dendrobium chrysanthum* Wall. ex Lindl. Acta Physiologiae Plantarum, 35, 2311–2322. 10.1007/s11738-013-1268-z

[ece37417-bib-0027] Hobbie, E. A. , & Agerer, R. (2010). Nitrogen isotopes in ectomycorrhizal sporocarps correspond to belowground exploration types. Plant and Soil, 327, 71–83. 10.1007/s11104-009-0032-z

[ece37417-bib-0028] Hobbie, E. A. , & Högberg, P. (2012). Nitrogen isotopes link mycorrhizal fungi and plants to nitrogen dynamics. New Phytologist, 196, 367–382. 10.1111/j.1469-8137.2012.04300.x 22963677

[ece37417-bib-0029] Hobbie, E. A. , Jumpponen, A. , & Trappe, J. (2005). Foliar and fungal ^15^N:^14^N ratios reflect development of mycorrhizae and nitrogen supply during primary succession: Testing analytical models. Oecologia, 146, 258–268. 10.1007/s00442-005-0208-z 16096847

[ece37417-bib-0030] Hobbie, J. E. , & Hobbie, E. A. (2006). ^15^N in symbiotic fungi and plants estimates nitrogen and carbon flux rates in arctic tundra. Ecology, 87, 816–822. 10.2307/20069010 16676524

[ece37417-bib-0031] Hodge, E. A. (2004). The plastic plant: Root responses to heterogeneous supplies of nutrients. New Phytologist, 162, 9–24. 10.1111/j.1469-8137.2004.01015.x

[ece37417-bib-0032] Houlton, B. Z. , Sigman, D. M. , Schuur, E. A. G. , & Hedin, L. O. (2007). A climate‐driven switch in plant nitrogen acquisition within tropical forest communities. Proceedings of the National Academy of Sciences of the United States of America, 104, 8902–8906. 10.1073/pnas.0609935104 17502607 PMC1885600

[ece37417-bib-0033] Jackson, R. B. , & Caldwell, M. M. (1996). Integrating resource heterogeneity and plant plasticity: Modelling nitrate and phosphate uptake in a patchy soil environment. Journal of Ecology, 84, 891–903. 10.2307/2960560

[ece37417-bib-0034] Jiang, J. , Moore, J. A. , Priyadarshi, A. , & Classen, A. T. (2017). Plant‐mycorrhizal interactions mediate plant community coexistence by altering resource demand. Ecology, 98, 187–197. 10.1002/ecy.1630 28052388

[ece37417-bib-0035] Johnson, N. C. (2010). Resource stoichiometry elucidates the structure and function of arbuscular mycorrhizas across scales. New Phytologist, 185, 631–647. 10.1111/j.1469-8137.2009.03110.x 19968797

[ece37417-bib-0036] Johnson, N. C. , Angelard, C. , Sanders, I. R. , & Kiers, E. T. (2013). Predicting community and ecosystem outcomes of mycorrhizal responses to global change. Ecology Letters, 16, 140–153. 10.1111/ele.12085 23679013

[ece37417-bib-0037] Johnson, N. C. , Rowland, D. L. , Corkidi, L. , Egerton‐Warburton, L. M. , & Allen, E. B. (2003). Nitrogen enrichment alters mycorrhizal allocation at five mesic to semiarid grasslands. Ecology, 84, 1895–1908.10.1890/0012‐9658(2003)084[1895:NEAMAA]2.0.CO;2

[ece37417-bib-0038] Keyimu, M. , Li, Z. S. , Wu, X. C. , Fu, B. J. , Liu, G. H. , Shi, S. L. , Fan, Z. X. , & Wang, X. C. (2020). Recent decline of high altitude coniferous growth due to thermo‐hydraulic constrains: Evidence from the Miyaluo Forest Reserve, Western Sichuan Plateau of China. Dendrochronologia, 63, 125751. 10.1016/j.dendro.2020.125751

[ece37417-bib-0039] Koele, N. , Dickie, I. A. , Oleksyn, J. , Richardson, S. J. , & Reich, P. B. (2012). No globally consistent effect of ectomycorrhizal status on foliar traits. New Phytologist, 196, 845–852. 10.1111/j.1469-8137.2012.04297.x 22966750

[ece37417-bib-0040] Köhle, J. , Yang, N. , Pena, R. , Rahavan, V. , Polle, A. , & Meier, I. C. (2018). Ectomycorrhizal fungal diversity increases phosphorus uptake efficiency of European beech. New Phytologist, 220, 1200–1210. 10.1111/nph.15208 29770963

[ece37417-bib-0041] Ladd, J. N. , & Butler, J. H. A. (1972). Short‐term assays of soil proteolytic enzyme activities using proteins and dipeptide derivatives as substrates. Soil Biology and Biochemistry, 4, 19–30. 10.1016/0038-0717(72)90038-7

[ece37417-bib-0042] Lande, R. (1996). Statistics and partitioning of species diversity, and similarity among multiple communities. Oikos, 76, 5–13. 10.2307/3545743

[ece37417-bib-0043] Landis, F. C. , & Fraser, L. H. (2008). A new model of carbon and phosphorus transfers in arbuscular mycorrhizas. New Phytologist, 177, 466. 10.1111/j.1469-8137.2007.02268.x 18028302

[ece37417-bib-0044] Li, Y. , Sun, D. , Li, D. , Xu, Z. , Zhao, C. , Lin, H. , & Liu, Q. (2015). Effects of warming on ectomycorrhizal colonization and nitrogen nutrition of *Picea asperata* seedlings grown in two contrasting forest ecosystems. Scientific Reports, 5, 17546. 10.1038/srep17546 26655633 PMC4674696

[ece37417-bib-0045] Li, Z. S. , Keyimu, M. , Fan, Z. , & Wang, X. C. (2020). Climate sensitivity of conifer growth doesn’t reveal distinct low–high dipole along the elevation gradient in the Wolong National Natural Reserve, SW China. Dendrochronologia, 61, 125702. 10.1016/j.dendro.2020.125702

[ece37417-bib-0046] Li, Z. S. , Liu, G. H. , Fu, B. J. , Zhang, Q. B. , Ma, K. P. , & Neil, P. (2013). The growth‐ring variations of alpine shrub *Rhododendron przewalskii* reflect regional climate signals in the alpine environment of Miyaluo Town in Western Sichuan Province, China. Acta Ecologica Sinica, 33, 23–31. 10.1016/j.chnaes.2012.12.004

[ece37417-bib-0047] Lilleskov, E. , Hobbie, E. , & Horton, T. (2011). Conservation of ectomycorrhizal fungi: Exploring the linkages between functional and taxonomic responses to anthropogenic N deposition. Fungal Ecology, 4, 174–183. 10.1016/j.funeco.2010.09.008

[ece37417-bib-0048] Liu, G. F. , Freschet, G. T. , Pan, X. , Cornelissen, J. H. C. , Li, Y. , & Dong, M. (2010). Coordinated variation in leaf and root traits across multiple spatial scales in Chinese semi‐arid and arid ecosystems. New Phytologist, 188, 543–553. 10.1111/j.1469-8137.2010.03388.x 20649915

[ece37417-bib-0049] Lõhmus, K. , Truu, M. , Truu, J. , Ostonen, I. , Kaar, E. , Vares, A. , Uri, V. , Alam, S. , & Kanal, A. (2006). Functional diversity of culturable bacterial communities in the rhizosphere in relation to fine‐root and soil parameters in alder stands on forest, abandoned agricultural, and oil‐shale mining areas. Plant and Soil, 283, 1–10. 10.1007/s11104-005-2509-8

[ece37417-bib-0050] Magyar, G. , Kun, Á. , Oborny, B. , & Stuefer, J. F. (2007). Importance of plasticity and decision‐making strategies for plant resource acquisition in spatio‐temporally variable environments. New Phytologist, 174, 182–193. 10.1111/j.1469-8137.2007.01969.x 17335508

[ece37417-bib-0051] Matsuda, Y. , & Hijii, N. (2004). Ectomycorrhizal fungal communities in an *Abiea firma* forest, with special reference to ectomycorrhizal associations between seedlings and mature trees. Canadian Journal of Botany, 83, 822–829. 10.1139/b04-065

[ece37417-bib-0052] Merrild, M. P. , Ambus, P. , Rosendahl, S. , & Jakobsen, I. (2013). Common arbuscular mycorrhizal networks amplify competition for phosphorus between seedlings and established plants. New Phytologist, 200, 229–240. 10.1111/nph.12351 23738787

[ece37417-bib-0053] Michelsen, A. , Schmidt, I. K. , Jonasson, S. , Quarmby, C. , & Sleep, D. (1996). Leaf ^15^N abundance of subarctic plants provides field evidence that ericoid, ectomycorrhizal and non‐ and arbuscular mycorrhizal species access different sources of soil nitrogen. Oecologia, 105, 53–63. 10.1007/BF00328791 28307122

[ece37417-bib-0054] Mitchell, K. (2007). Quantitative analysis by the point‐centered quarter method. PhD thesis. Hobart and William Smith Colleges.

[ece37417-bib-0055] Näsholm, T. , Högberg, P. , Franklin, O. , Metcalfe, D. , Keel, S. G. , Campbell, C. , Hurry, V. , Linder, S. , & Högberg, M. N. (2013). Are ectomycorrhizal fungi alleviating or aggravating nitrogen limitation of tree growth in boreal forests? New Phytologist, 198, 214–221. 10.1111/nph.12139 23356503

[ece37417-bib-0056] Nehls, U. , & Plassard, C. (2018). Nitrogen and phosphate metabolism in ectomycorrhizas. New Phytologist, 220, 1047–1058. 10.1046/j.1469-8137.1999.00513.x 29888395

[ece37417-bib-0057] Nelson, D. W. , & Sommers, L. E. (1982). Total carbon, organic carbon, and organic matter. In A. L. Page , R. H. Miller , & D. R. Keeney (Eds.), Methods of Soil Analysis (pp. 101–129). American Society of Agronomy and Soil Science Society of American.

[ece37417-bib-0058] New, M. , Hulme, M. , & Jones, P. (2000). Representing twentieth‐century space‐time climate variability. Part II: Development of 1901–96 monthly grids of terrestrial surface climate. Journal of Climate, 13, 2217–2238. 10.1175/1520-0442(1999)0122.0.CO

[ece37417-bib-0059] Ostonen, I. , Helmisaari, H. S. , Borken, W. , Tedersoo, L. , Kukumagi, M. , Bahram, M. , Lindroos, A.‐J. , Nojd, P. , Uri, V. , Merila, P. , Asi, E. , & Lõhmus, K. (2011). Fine root foraging strategies in Norway spruce forests across a European climate gradient. Global Change Biology, 17, 3620–3632. 10.1111/j.1365-2486.2011.02501.x

[ece37417-bib-0060] Ostonen, I. , Lõhmus, K. , Helmisaari, H. S. , Truu, J. , & Meel, S. (2007). Fine root morphological adaptations in Scots pine, Norway spruce and silver birch along a latitudinal gradient in boreal forests. Tree Physiology, 27, 1627–1634. 10.1093/treephys/27.11.1627 17669752

[ece37417-bib-0061] Ostonen, I. , Tedersoo, L. , Suvi, T. , & Lõhmus, K. (2009). Does a fungal species drive ectomycorrhizal root traits in *Alnus* spp.? Canadian Journal of Forest Research, 39, 1787–1796. 10.1139/X09-093

[ece37417-bib-0062] Plassard, C. , & Dell, B. (2010). Phosphorus nutrition of mycorrhizal trees. Tree Physiology, 30, 1129–1139. 10.1093/treephys/tpq063 20631011

[ece37417-bib-0063] Pritsch, K. , & Garbaye, J. (2011). Enzyme secretion by ECM fungi and exploitation of mineral nutrients from soil organic matter. Annals of Forest Science, 68, 25–32. 10.1007/s13595-010-0004-8

[ece37417-bib-0064] Read, D. J. (1991). Mycorrhizas in ecosystems. Cellular and Molecular Life Sciences, 47, 376–391. 10.1007/BF01972080

[ece37417-bib-0065] Rosinger, C. , Sandén, H. , Matthews, B. , Mayer, M. , & Godbold, D. L. (2018). Patterns in ectomycorrhizal diversity, community composition, and exploration types in European beech, pine, and spruce forests. Forests, 9, 445. 10.3390/f9080445

[ece37417-bib-0066] Schinner, F. , Ohlinger, R. , Kandeler, E. , & Margesin, R. (1996). Methods in soil biology. Springer.

[ece37417-bib-0067] Smith, S. E. , Jakobsen, I. , Grönlund, M. , & Smith, F. A. (2011). Roles of arbuscular mycorrhizas in plant phosphorus nutrition: Interactions between pathways of phosphorus uptake in arbuscular mycorrhizal roots have important implications for understanding and manipulating plant phosphorus acquisition. Plant Physiology, 156, 1050–1057. 10.1104/pp.111.174581 21467213 PMC3135927

[ece37417-bib-0068] Smith, S. , & Read, D. (2008). Mycorrhizal symbiosis. Academic Press.

[ece37417-bib-0069] Steven, A. T. , Rygiewcz, P. T. , & Edmonds, R. L. (2004). Patterns of nitrogen and carbon stable isotope ratios in macrofungi, plants and soils in two old‐growth conifer forests. New Phytologist, 164, 317–335. 10.1111/j.1469-8137.2004.01162.x 33873563

[ece37417-bib-0070] Taylor, A. H. , Jang, S. W. , Zhao, L. J. , Liang, C. P. , Miao, C. J. , & Huang, J. Y. (2006). Regeneration patterns and tree species coexistence in old‐growth *Abies‐Picea* forests in southwestern China. Forest Ecology and Management, 223, 303–317. 10.1016/j.foreco.2005.11.010

[ece37417-bib-0071] Tedersoo, L. , Naadel, T. , Bahram, M. , Pritsch, K. , Buegger, F. , Leal, M. , Kõljalg, U. , & Põldmaa, K. (2012). Enzymatic activities and stable isotope patterns of ectomycorrhizal fungi in relation to phylogeny and exploration types in an afrotropical rain forest. New Phytologist, 195, 832–843. 10.1111/j.1469-8137.2012.04217.x 22758212

[ece37417-bib-0072] Tessier, J. T. , & Raynal, D. J. (2003). Use of nitrogen to phosphorus ratios in plant tissue as an indicator of nutrient limitation and nitrogen saturation. Journal of Applied Ecology, 40, 523–534. 10.1046/j.1365-2664.2003.00820.x

[ece37417-bib-0073] Tjoelker, M. G. , Craine, J. M. , Wedin, D. , Reich, P. B. , & Tilman, D. (2005). Linking leaf and root trait syndromes among 39 grassland and savannah species. New Phytologist, 167, 493–508. 10.1111/j.1469-8137.2005.01428.x 15998401

[ece37417-bib-0074] Toljander, J. F. , Eberhardt, U. , Toljander, Y. K. , Paul, L. R. , & Taylor, A. F. S. (2006). Species composition of an ectomycorrhizal fungal community along a local nutritional gradient. New Phytologist, 170, 873–884. 10.1111/j.1469-8137.2006.01718.x 16684245

[ece37417-bib-0075] Treseder, K. K. (2004). A meta‐analysis of mycorrhizal responses to nitrogen, phosphorus, and atmospheric CO_2_ in field studies. New Phytologist, 164, 347–355. 10.1111/j.1469-8137.2004.01159.x 33873547

[ece37417-bib-0076] Treseder, K. K. , & Allen, M. F. (2002). Direct nitrogen and phosphorus limitation of arbuscular mycorrhizal fungi: A model and field test. New Phytologist, 155(3), 507–515. 10.1046/j.1469-8137.2002.00470.x 33873310

[ece37417-bib-0077] Vandenkoornhuyse, P. , Ridgway, K. P. , Watson, I. J. , Fitter, A. H. , & Young, J. P. (2003). Co‐existing grass species have distinctive arbuscular mycorrhizal communities. Molecular Ecology, 12, 3085–3095. 10.1046/j.1365-294X.2003.01967.x 14629388

[ece37417-bib-0078] Vitousek, P. M. , Porder, S. , Houlton, B. Z. , & Chadwick, O. A. (2010). Terrestrial phosphorus limitation: Mechanisms, implications, and nitrogen–phosphorus interactions. Ecological Applications, 20, 1–5. 10.1890/08-0127.1 20349827

[ece37417-bib-0079] Walker, T. W. , & Syers, J. K. (1976). The fate of phosphorus during pedogenesis. Geoderma, 15, 1–19. 10.1016/0016-7061(76)90066-5

[ece37417-bib-0080] Wang, L. , Mou, P. P. , & Jones, R. H. (2006). Nutrient foraging via physiological and morphological plasticity in three plant species. Canadian Journal of Forest Research, 36, 164–173. 10.1139/x05-239

[ece37417-bib-0081] Xu, Y. , Gao, X. J. , Shen, Y. , Xu, C. H. , Shi, Y. , & Giorgi, F. (2009). A daily temperature dataset over China and its application in validating a RCM simulation. Advances in Atmospheric Sciences, 26(4), 763–772. 10.1007/s00376-009-9029-z

[ece37417-bib-0082] Zavišić, A. , Nassal, P. , Yanga, N. , Heuck, C. , Spohn, M. , Marhan, S. , Pena, R. , Kandeler, E. , & Polle, A. (2016). Phosphorus availabilities in beech (*Fagus sylvatica* L.) forests impose habitat filtering on ectomycorrhizal communities and impact tree nutrition. Soil Biology and Biochemistry, 98, 127–137. 10.1016/j.soilbio.2016.04.006

[ece37417-bib-0083] Zhang, W. R. (1983). The forest soils of Wolong Natural Reserve and its vertical zonalties distribution. Scientia Silvae Sinicae, 19(3), 254–268.

[ece37417-bib-0084] Zhang, Z. , Li, N. , Xiao, J. , Zhao, C. , Zou, T. T. , Li, D. D. , Liu, Q. , & Yin, H. (2018). Changes in plant nitrogen acquisition strategies during the restoration of spruce plantations on the eastern Tibetan Plateau, China. Soil Biology and Biochemistry, 119, 50–58. 10.1016/j.soilbio.2018.01.002

[ece37417-bib-0085] Zhang, Z. L. , Yuan, Y. S. , Liu, Q. , & Yin, H. J. (2019). Plant nitrogen acquisition from inorganic and organic sources via root and mycelia pathways in ectomycorrhizal alpine forests. Soil Biology and Biochemistry, 136, 1–9. 10.1016/j.soilbio.2019.06.013

[ece37417-bib-0086] Zhao, T. B. , Guo, W. D. , & Fu, C. B. (2008). Calibrating and evaluating reanalysis surface temperature error by topographic correction. Journal of Climate, 21(6), 1440–1446. 10.1175/2007JCLI1463.1

[ece37417-bib-0087] Zhao, Z. J. , Eamus, D. , Yu, Q. , Li, Y. , Yang, H. W. , & Li, J. Q. (2012). Climate constraints on growth and recruitment patterns of *Abies faxoniana* over altitudinal gradients in the Wanglang Natural Reserve, eastern Tibetan Plateau. Australian Journal of Botany, 60, 602–614. 10.1071/BT12051

